# Effectiveness of psychoeducational support on quality of life in early-stage breast cancer patients: a systematic review and meta-analysis of randomized controlled trials

**DOI:** 10.1007/s11136-013-0460-3

**Published:** 2013-07-24

**Authors:** Ayako Matsuda, Kazue Yamaoka, Toshiro Tango, Tomohiro Matsuda, Hiroshi Nishimoto

**Affiliations:** 1 Surveillance Division, Center for Cancer Control and Information Services, National Cancer Center, 5-1-1 Tsukiji, Chuo-ku, Tokyo 104-0045 Japan; 2 Graduate School of Public Health, Teikyo University, 2-11-1 Kaga, Itabashi-ku, Tokyo 173-8605 Japan; 3 Center for Medical Statistics, 2-9-6 Higashi-Shinbashi, Minato-ku, Tokyo 105-0021 Japan

**Keywords:** Quality of life, Meta-analysis, Breast cancer, Psychosocial support, Psychoeducational support

## Abstract

**Purpose:**

Most breast cancer patients receive psychosocial support interventions. However, the effectiveness of these interventions has not yet been clarified. Quality of life (QOL) was an important construct that should be considered when assessing these interventions. The purpose was to evaluate the effectiveness of psychosocial and especially psychoeducational support interventions for early-stage breast cancer patients since the follow-up was bound up to 6 months after finishing the intervention.

**Methods:**

We conducted a systematic review and meta-analysis to identify randomized controlled trials with early-stage breast cancer patients receiving psychosocial (psychoeducational and other) support in which QOL was measured as a treatment outcome. We compared mean differences at less than 6 months post-intervention with a control group. The primary outcome was Global Health Status/QOL scale (Global QOL), and secondary outcomes were the subscales of QOL.

**Results:**

No significant effect was observed for Global QOL; however, individuals receiving psychosocial support scored higher on the Breast Cancer Symptoms subscale. For psychoeducational support in the psychosocial support, significant effect was observed on the Emotional subscale.

**Conclusions:**

Our analysis strengthens the evidence of the effectiveness of psychosocial support in improving breast cancer symptoms and psychoeducational support in improving emotional well-being within 6 months post-intervention.

## Introduction

Breast cancer is the most frequently diagnosed cancer among women in global cancer statistics [1]. Incidence rates are much higher in more developed countries, and observed improvements in breast cancer survival over recent decades have been attributed to the systematic use of adjuvant therapies [2]. However, breast cancer patients may experience many manifestations resulting from the primary disease and/or treatment for the disease, and face issues related to simultaneously dealing with a multitude of physical and psychological symptoms [3].

Interventions after primary treatment for breast cancer should have several aims. Psychosocial support can provide assistance and encouragement to individuals with physical or emotional disabilities. There are many research papers on the effectiveness of psychosocial interventions on quality of life (QOL). However, most of the interventions aimed at physical symptoms have beneficial effects on QOL at varying follow-up periods [4, 5]. Results of a meta-analysis revealed that behavioral techniques and physical exercise improve psychosocial functioning and QOL [6]. The psychosocial aspect includes interventions described as psychological, psychotherapeutic, psychoeducational, or psychosocial [7]. Psychosocial constructs that have the strongest association with QOL are stress, affect, and cognitive appraisal [8]. Although to improve emotional well-being is important, the effectiveness of interventions other than exercise has not been clarified in QOL. The psychosocial interventions are wide ranging (e.g., psychoeducational support, cognitive behavioral therapy, and emotional expression). One of the most effective psychosocial approaches to cancer patients is psychoeducation [9]. The psychoeducational interventions in psychosocial interventions specifically address emotional concerns arising from the distress that can be caused by being overwhelmed or confused [7]. We considered that it was important to evaluate a focus on psychoeducational interventions in psychosocial interventions. In many studies, the majority of the women with breast cancer reported needing increased educational support [10]. Recent meta-analysis by Cochrane review examined the effect psychoeducational intervention on QOL as a part of psychosocial support interventions for cancer patients, and the psychoeducational interventions produced small positive significant effects on QOL by the result of only one study [7].

The purpose in this study was to evaluate the effectiveness of psychosocial and especially psychoeducational support interventions to improve QOL for early-stage breast cancer patients since the follow-up was bound up to 6 months after finishing the intervention. We conducted a meta-analysis to evaluate the effectiveness of psychosocial support interventions other than exercise for early-stage breast cancer patients, paying particular attention to their QOL. Additionally, we considered QOL in psychosocial support that was subdivided into psychoeducational or other psychosocial support. The results of this study are expected to provide useful information for early-stage breast cancer patients receiving psychosocial support, taking into consideration QOL.

## Materials and methods

### Study design

This study is a systematic literature review and meta-analysis.

### Search for trials

Trials were identified by an electronic search of the PubMed database and the Cochrane Central Register of Controlled Trials (CENTRAL) database. Search terms used were “quality of life” [MeSH Terms] AND “breast neoplasms” [MeSH Terms] AND “social support,” with searches limited to publications of randomized controlled trials (RCTs) in humans.

### Selection of trials

We conducted a literature search using the Cochrane Database and PubMed database (data from September 1988 to January 2012) to identify RCTs on breast cancer interventions in which QOL was measured as a treatment outcome.

Trials were eligible for inclusion in the meta-analysis if they compared an intervention group receiving psychosocial support with a control group in early-stage breast cancer patients and if they reported QOL data using a QOL questionnaire. All inclusion and exclusion criteria for selection of trials are shown in Table 1. The trials were then hand searched according to these criteria.

**Table 1:** Inclusion/exclusion criteria for selected trials

Inclusion criteria	Exclusion criteria
Randomized controlled clinical trials	Studies including patients with metastatic or advanced stage cancer
Studies on breast cancer	Studies including patients with psychiatric problems
Studies comparing a group receiving social support with a control group^a^	Not an intervention study
	Intervention studies that included exercise as social support
	Studies not reporting adequate information on the randomization process in the “Materials and methods” or “Results” section
	Studies not reporting health-related quality of life (HRQOL) data using a QOL questionnaire

^a^Social support systems provide assistance and encouragement to individuals with physical or emotional disabilities so they can better cope

**Table 2:** Summary of characteristics in selected RCTs

Author	Years	Country	Follow-up (month)	*n* (intervention/control) (610/549)	Social support intervention		Period of the intervention (months)	Sessions
Sandgren [20]^a^	2003	USA	5	78/55	Psychoeducational support	Health education, emotional expression	3	6
Meneses [18]	2007	USA	3, 6	125/131		Breast Cancer Education Intervention (BCEI) psychoeducational support	6	8
Beatty [15]	2010	Australia	3, 6	25/24		Self-help workbook (each chapter containing educational information on common medical and psychosocial issues)	6	
Gustafson [21]	2001	USA	2, 5	121/125	Other psychosocial Support	Comprehensive Health Enhancement Support System (CHESS)	5	
Sandgren [20]^a^	2003	USA	5	89/55		Health education, emotional expression	3	6
Owen [19]	2005	USA	3	62/62		Online coping program	3	
Gellaitry [17]	2010	UK	1, 3, 6	38/42		Writing intervention (emotional disclosure, cognitive appraisal, benefit finding, looking to the future)	6	4
Salzer [16]	2010	USA	4, 12	50/26		Internet peer support	12	
Cousson-Gelie [14]	2011	France	1^b^	22/29		Specific intervention (a specific psychosocial intervention)	1	8

^a^This study compared separately the effectiveness of health education and emotional expression interventions to that of standard care

^b^After eight sessions (or 1 month for the control group)

### Data extraction

Among the QOL scales, we focused on Global Health Status/QOL scale (Global QOL) and the 5 subscales [Breast Cancer Symptoms, Physical, Emotional (Psychological), Social, and Functional] that were most often assessed across studies. We only used QOL data collected at baseline and less than 6 months after the start of intervention to observe the effectiveness of interventions. In the assessment of risk of bias in included studies, the review authors worked to assess the methodological quality (random allocation, allocation concealment, blinding, and loss to follow-up) [7] of each selected study.

### Statistical analysis

We compared mean differences in Global QOL and subscales scores at less than 6 months post-intervention in early-stage breast cancer patients receiving psychosocial support (intervention group) and no such support (control group). The control group received normal care. These differences between groups were treated as effect sizes in our meta-analysis. After that, psychosocial support was then subdivided into psychoeducational or other psychosocial support.

Overall estimates were examined using a random-effects model (DerSimonian–Laird method) [11] and a fixed-effects model (general variance-based method). The χ^2^ test was used to assess heterogeneity among trials. Considering that the fixed-effects model is useful only under conditions of homogeneity and that the power of statistical tests of heterogeneity is low, we planned to use the random-effects model as the primary method irrespective of the test result for heterogeneity. A fixed-effects model was used for sensitivity analysis. S-plus and R programs [12, 13] were used for the estimation of the random-effects and fixed-effects models. A favorable outcome from psychosocial support (psychoeducational or other psychosocial support) was reflected when the mean for QOL was greater for the intervention group than for the control group.

All scale scores linearly transformed to a 0–100 scale, with higher scores indicating more positive outcomes. In this study, a statistical test with a *p* value less than 0.05 (two-side) was considered significant.

## Results

### Study characteristics

**Fig. 1:**
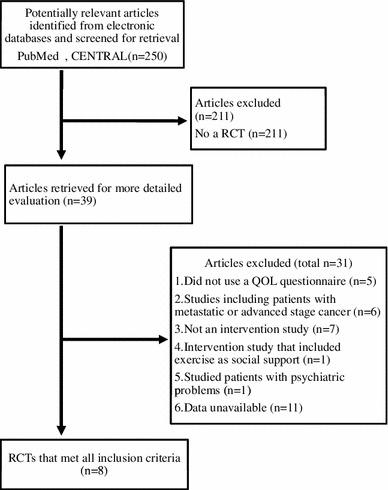
Systematic review flow chart. *n* number of articles, *CENTRAL* Cochrane Central Register of Controlled Trials, *RCT* randomized controlled trial, *QOL* quality of life

The process of study selection is illustrated in Fig. 1. Potentially relevant articles (*n* = 250) were identified from the search of the electronic databases. After this initial screening, no RCT articles (*n* = 211) were excluded leaving 39 articles. We identified 8 trials [14–21] using the exclusion criteria shown in Table 1. The study by Sandgren et al. [20] used two types of psychosocial support interventions (health education and emotional expression). Each was treated as an independent intervention. The trials reported QOL data using the European Organization for Research and Treatment of Cancer Core Questionnaire (EORTC QLQ-C30) [22], Functional Assessment of Cancer Therapy–Breast (FACT-B) [23], or Quality of Life–Cancer Survivors (QOL-CS) [24] (Table 3). The EORTC QLQ-C30 and FACT-General (FACT-G), probably the two most widely used oncological QOL instruments, were subjected to equating [25]. In both the FACT-G and QOL-CS developed by Cella, there is a moderate to strong correlation between associated subscales including QOL-CS Physical to FACT Physical, QOL-CS Psychological to FACT Emotional, QOL-CS Social to FACT Social, and overall QOL-CS to the FACT-G [26]. The FACT-Breast (FACT-B) that indicates Global QOL in breast cancer patients comprises the FACT-G plus the Breast Cancer Symptoms (BCS) subscale, which contains items specific to QOL in these patients. The standard deviation was taken from a study of the reliability and validity of the FACT-B [23] and QOL-CS [24] if it was not reported in the literature. In trials by Meneses et al. [27], we removed this study because the survivors in this study were a subset of the larger group study [18]. We considered the data in trials by Park et al. [28] not appropriate because the standard deviation of the FACT-B total was approximately similar to that of the subscale scores in that study [28] and was considerably smaller compared with that of other trials [14–21]. Characteristics of the selected 8 trials (Table 2) and QOL scores of these studies (Table 4) have been summarized. In total, 1,159 patients were randomly selected, with 610 receiving psychosocial support: psychoeducational support (228 patients) and other psychosocial support (382 patients). A total of 549 patients were in control groups. In the selected 8 trials, the methodological quality was insufficient information to assess low or high risk of bias.

**Table 3:** Summary of scores on QOL at less than 6 months post-intervention for selected RCTs

Authors	Questionnaires	Global QOL		BCS		Physical		Emotional (psychological)		Social		Functional	
		Intervention	Control	Intervention	Control	Intervention	Control	Intervention	Control	Intervention	Control	Intervention	Control
		Mean (SD)	Mean (SD)	Mean (SD)	Mean (SD)	Mean (SD)	Mean (SD)	Mean (SD)	Mean (SD)	Mean (SD)	Mean (SD)	Mean (SD)	Mean (SD)
Sandgren [20]^a^	FACT-B	77.6 (12.7)	77.6 (10.9)	67.2 (18.1)	65.9 (15.9)	80.5 (18.9)	80.1 (14.8)	84.5 (16.0)	82.6 (14.0)	75.8 (10.7)	77.3 (10.9)	80.6 (18.9)	83.0 (14.8)
Meneses [18]^b^	QOL-BC	71.7 (13.1)	69.0 (13.1)			81.8 (16.7)	80.3 (16.7)	67.5 (15.6)	62.0 (15.6)	73.5 (19.8)	71.0 (19.8)		
Beatty [15]	EORTC QLQ C30	69.0 (13.6)	72.2 (13.6)										
Gustafson [21]	FACT-B			67.6 (18.2)	64.7 (18.2)			76.3 (12.6)	75.3 (12.6)	75.8 (18.7)	74.7 (18.7)	70.4 (23.0)	69.9 (23.0)
Sandgren [20]^a^	FACT-B	79.0 (11.4)	77.6 (10.9)	69.5 (15.1)	65.9 (15.9)	79.6 (18.8)	80.0 (14.8)	84.8 (15.6)	82.6 (14.0)	77.3 (12.1)	77.3 (10.9)	83.5 (18.8)	83.0 (14.8)
Owen [19]	FACT-B	59.9 (7.3)	57.6 (8.0)	70.8 (13.3)	66.4 (17.5)			59.4 (9.4)	55.1 (13.0)				
Gellaitry [17]	FACT-B	76.1 (13.8)	75.0 (14.8)										
Salzer [16]^b^	FACT-B	70.5 (14.5)	76.9 (14.5)										
Cousson-Gelie [14]	EORTC QLQ C30	68.1 (16.1)	69.4 (17.6)			82.0 (15.8)	86.6 (13.2)	74.2 (19.8)	83.9 (18.5)	82.6 (21.4)	90.7 (18.1)		

All scale scores linearly transformed to a 0–100 scale, with higher scores indicating more positive outcomes

*EORTC QLQ*-*C30* European Organization for Research and Treatment of Cancer Core Questionnaire, *FACT*-*B* Functional Assessment of Cancer Therapy–Breast, *QOL*-*BC* Quality of Life–Breast Cancer Survivors, *Global QOL* FACT-B; FACT-B total, QOL-BC; Overall QOL, EORTC QLQ C30; Global, *BCS* breast cancer symptoms

^a^This study compared separately the effectiveness of health education and emotional expression interventions to that of standard care

^b^The standard deviation (SD) was taken from “Reliability and validity of the Functional Assessment of Cancer Therapy-Breast quality-of-life instrument” [23] or “Quality of life in long-term cancer survivors”[24]

**Table 4:** Estimates of effects per intervention

Outcome measure	All (psychoeducational support + other psychosocial support)		Psychoeducational support^b^		Other psychosocial support	
	Mean difference (95 % CI)	*p* value	Mean difference (95 % CI)	*p* value	Mean difference (95 % CI)	*p* value
Global QOL	0.968 (−0.721 to 2.656)	0.261	1.008 (−1.775 to 3.790)	0.478	0.620 (−1.957 to 3.197)	0.637
BCS^a^	3.110 (0.504 to 5.716)	0.019	–	–	3.540 (0.641 to 6.439)	0.017
Physical	0.124 (−2.621 to 2.870)	0.929	1.149 (−2.228 to 4.527)	0.505	−1.870 (−6.583 to 2.842)	0.453
Emotional (psychological)	2.360 (−0.195 to 4.915)	0.070	4.167 (0.760 to 7.574)	0.017	1.338 (−2.160 to 4.836)	0.453
Social	−0.088 (−2.155 to 1.979)	0.934	0.185 (−3.686 to 4.055)	0.926	−0.269 (−3.536 to 2.998)	0.872
Functional	−0.427 (−3.808 to 2.954)	0.805	–	–	0.500 (−3.600 to 4.599)	0.811

^a^ *BCS* breast cancer symptoms

^b^The BCS and Functional Well-Being subscale were each reported in only one study

### QOL

**Fig. 2:**
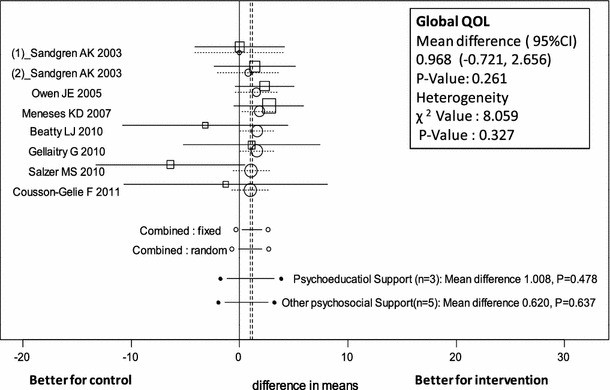
Forest plot of mean difference in Global QOL scores of patients receiving psychosocial support (psychoeducational and other psychosocial support) with 95 % CI for each study, overall for several models (*circles* represent cumulative meta-analysis)

**Fig. 3:**
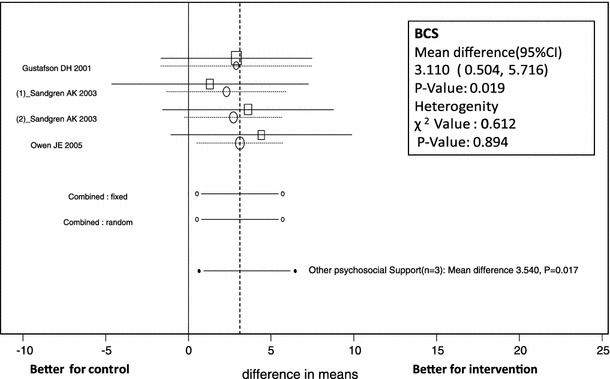
Forest plot of mean difference in Breast Cancer Symptoms (BCS) subscale scores of patients receiving psychosocial support with 95 % CI for each study, overall for several models (*circles* represent cumulative meta-analysis)

In all trials, analysis showed no evidence of heterogeneity among studies for Global QOL (*p* = 0.327). The mean difference in Global QOL was estimated as 0.968 (95 % CI −0.721 to 2.656, *p* = 0.261) by the random-effects model and did not differ significantly between the psychosocial support intervention group and control group (Table 4; Fig. 2). In the 5 subscales, individuals receiving support scored higher on the BCS (mean difference 3.110, 95 % CI 0.504–5.716, *p* = 0.019) subscale of QOL (Table 4; Fig. 3).

### Psychoeducational support and other psychosocial support

**Fig. 4:**
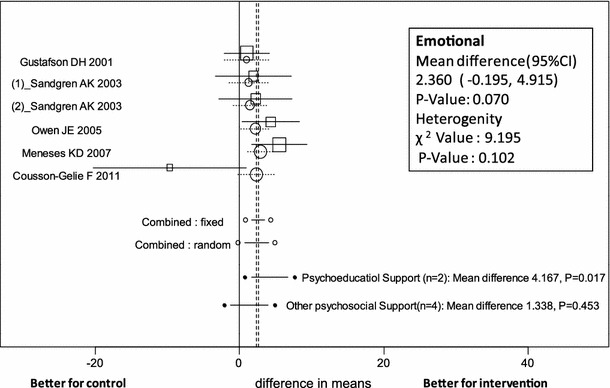
Forest plot of mean difference in Emotional subscale scores of patients receiving psychosocial support (psychoeducational and other psychosocial support) with 95 % CI for each study, overall for several models (*circles* represent cumulative meta-analysis)

Analysis of Global QOL related to psychoeducational support in 3 trials showed no evidence of heterogeneity among studies (*p* = 0.295). The mean difference in Global QOL was estimated as 1.008 (95 % CI −1.775 to 3.790, *p* = 0.478) by the random-effects model, not a statistically significant difference between psychoeducational support intervention and control groups (Table 4; Fig. 2). For the subscales, individuals receiving support scored highest on the Emotional subscale (mean difference 4.167, 95 % CI 0.760–7.574, *p* = 0.017) of QOL (Table 4; Fig. 4). The BCS and Functional Well-Being subscale were each reported in only one study.

Regarding Global QOL related to other types of psychosocial support in 5 trials, analysis showed no evidence of heterogeneity among studies (*p* = 0.230). The mean difference in Global QOL was estimated as 0.620 (95 %CI −1.957 to 3.197, *p* = 0.637) by the random-effects model, not a statistically significant difference between other psychosocial support interventions and control groups (Table 4; Fig 2). For the subscales, individuals receiving support scored highest on the BCS items (mean difference 3.540, 95 % CI 0.641–6.439, *p* = 0.017) of QOL (Table 4; Fig 3).

## Discussion

Our meta-analysis provided the evidence that the psychosocial support was effective in improving breast cancer symptoms (mean difference 3.110, 95 % CI 0.504–5.716, *p* = 0.019) within 6 months post-intervention. The psychoeducational support in the psychosocial support was effective in increasing emotional well-being (mean difference 4.167, 95 % CI 0.760–7.574, *p* = 0.017). With regard to different types of psychosocial support, higher emotional well-being was reported within 6 months post-intervention as a result of psychoeducational support, but not other types of psychosocial support. We did not conduct a meta-analysis regarding psychoeducational support related to breast cancer symptoms because the scale of breast cancer symptoms was reported in only one study. However, we posit that psychoeducational support was effective in improving the symptoms because individuals receiving it scored higher on the BCS, indicating greater QOL specific to breast cancer [20].

A previous meta-analysis indicated that physical exercise interventions improve QOL in breast cancer patients and survivors [6]. In contrast, this meta-analysis showed that psychosocial support interventions did not provide a significant benefit in terms of improved Global QOL in early-stage breast cancer patients. The result of Cochrane review [7] showed similar result. Namely, no significant effects were observed for QOL, while the psychoeducational interventions produced small positive significant effects on QOL [7]. However, the result was based on only one study and they did not examine the effects on subscales of QOL. For the results of the subscales, we suggested the effectiveness of psychosocial support in improving breast cancer symptoms and psychoeducational support in improving emotional well-being. Psychosocial support is an important intervention for cancer patients because stress-related psychosocial factors can have an adverse effect on cancer outcomes [29]. Specially, psychoeducation was shown to cause positive changes in levels of adjustment to cancer in breast cancer patients [9]. Chan et al. [30] also described that psychoeducational intervention was a promising treatment for relieving the symptom cluster and each of the individually assessed symptoms. Furthermore, Rottmann et al. [31] found a significant association between education and self-efficacy that was a significant predictor of emotional well-being in breast cancer patients.

In this meta-analysis, Global QOL and subscale scores tended to improve as a result of psychosocial support interventions. Therefore, it is important to assess QOL of breast cancer patients who receive psychosocial support. QOL is increasingly recognized as a major end point in medical care [32]. The US Food and Drug Administration welcomes the opportunity to explore with investigators the use of QOL instruments in the design of cancer clinical trials [33]. Information regarding QOL is invaluable in understanding the full impact of treatment differences on patient outcomes [34], and enhanced understanding of patient QOL can help improve clinical care [35].

We speculate that there may be differences in the effectiveness of psychosocial support interventions based on how they are administered. In 3 studies about psychoeducational support [15, 18, 20], the interventions consisted of individual face-to-face education. In 3 studies in other psychosocial support group, the interventions consisted of computer support [16, 19, 21]. In this meta-analysis, only psychoeducational support was effective in improving emotional well-being within 6 months post-intervention. Interventions for early-stage breast cancer patients have frequently combined elements of psychoeducational and other psychosocial support, stress management techniques, and cognitive behavioral therapy [36, 37]. In a study by Grunfeld et al. [38], compilation of survivorship care plans and a psychoeducational session were undertaken in a pragmatic trial that was consistent with usual practices and feasible to implement within time and human resource constraints. It is important to examine whether interventions need to combine psychoeducational, emotional, and physical support while considering timing of such interventions.

The measurement of outcomes is more complex in psychosocial research than in most drug-based studies and clinical measures; however, the increasing number of publications of psychosocial interventions indicates this is an area of huge interest [7]. In this meta-analysis, breast cancer symptoms and emotional well-being were improved by psychosocial support interventions, especially psychoeducational support within 6 months post-intervention. Future research should focus on evaluating their effectiveness related to long-term outcomes such as mortality and morbidity at follow-up [39]. In addition, based on a meta-analysis of the effectiveness of psychosocial interventions, a definite conclusion about whether such interventions prolong cancer survival seems premature [40].

### Strengths and limitations

To our knowledge, this is the first study employing a meta-analysis of RCT studies that has examined the effects of psychosocial support by classifying psychoeducational and emotional interventions for breast cancer patients and survivors. However, the psychoeducational training used was not uniform across studies. Strengths of our study include analyzing only RCTs and assessing the magnitude of effectiveness according to mean differences in QOL scores.

Certain limitations of this study should be considered. The first is publication bias. This analysis was confined to English-language articles, which could have contributed to such bias. Considering that the quality of studies on psychosocial support may be affected by many confounding biases, this limitation may be acceptable. Publication bias is always a concern in meta-analyses, and although the chance may be small, we cannot deny that possibility.

A second limitation is the variability in psychosocial support programs. Considering the heterogeneity, we used a random-effects model as the primary analysis and classified psychosocial support into two types: psychoeducational and other psychosocial support. Although the quality and content of psychosocial support programs varied, the results indicated that psychoeducational support was effective.

A third limitation is that this meta-analysis included a small number of subjects compared with previous studies [4–6] (610 patients vs. 549 controls in our study). However, some of the studies were pilot studies.

A fourth limitation is that some of the studies did not report any subscales scores. This could lead to conservative *p* values. However, our results suggested significant associations with some QOL measures.

Furthermore, even though our search method included a systematic review and added hand search, we could have inadvertently missed eligible studies. The results should be interpreted carefully considering a risk of bias across studies.

## Conclusions

Our analysis strengthens the evidence of the effectiveness of psychosocial support in improving breast cancer symptoms and psychoeducational support in improving emotional well-being within 6 months post-intervention. However, further long-term interventions may be needed to examine the effectiveness of other types of psychosocial support on improving the QOL of early-stage breast cancer patients.

In the future, research should focus on evaluating the effectiveness of psychosocial support interventions considering long-term outcomes and examine the influence of such interventions on survival time in cancer patients.
